# S100A12 is a promising biomarker in papillary thyroid cancer

**DOI:** 10.1038/s41598-020-58534-1

**Published:** 2020-02-03

**Authors:** Xiaojie Wang, Zhenxiang Sun, Wei Tian, Chenghao Piao, Xiaochen Xie, Jin Zang, Shiqiao Peng, Xiaohui Yu, Yiwei Wang

**Affiliations:** 1 Department of anatomy, Shenyang Medical College, Huanggu District, Shenyang City, Liaoning Province 110034 P.R. China; 2 Department of Radiology, The Second Affiliated Hospital of Shenyang Medical College, Shenyang City, Liaoning Province 110035 P.R. China; 3 Department of Endocrinology and Metabolism, Institute of Endocrinology, Liaoning Provincial Key Laboratory of Endocrine Diseases, The First Affiliated Hospital of China Medical University, China Medical University, Shenyang, Liaoning 110001 P.R. China

**Keywords:** Thyroid cancer, Oncogenes

## Abstract

S100A12 belongs to the S100 family and acts as a vital regulator in different types of tumors. However, the function of S100A12 in thyroid carcinoma has not yet been investigated. In this study, we analyzed the expression of S100A12 in human papillary thyroid cancer (PTC) samples and two PTC cell lines. In addition, we explored the effects of S100A12 on PTC cell progression *in vitro* and *in vivo*. Our results showed that S100A12 was significantly upregulated in PTC specimens. Moreover, silencing S100A12 markedly inhibited PTC cell proliferation, migration, invasion and cell cycle progression. In addition, knockdown of S100A12 significantly reduced the expression of CyclinD1, CDK4 and p-ERK in PTC cells. An *in vivo* study also showed that silencing S100A12 dramatically suppressed tumor cell growth and decreased Ki67 expression in a xenograft mouse model. This study provides novel evidence that S100A12 serves as an oncogene in PTC. Knockdown of S100A12 suppressed PTC cell proliferation, migration, and invasion and induced G0/G1 phase arrest via the inhibition of the ERK signaling pathway. Therefore, S100A12 may be a potent therapeutic target for PTC.

## Introduction

PTC is one of the most common endocrine malignant tumors, accounting for approximately 80% of thyroid carcinomas^1^. Although the use of several therapeutic methods for human PTC, such as surgery, chemotherapy and radiation, is continually increasing^2–4^, over 30% of patients present with a high tendency toward lymph node metastasis and recurrence within a decade^5^. Thus, discovering the mechanisms that mediate the invasion and migration of PTC is vital to the treatment of thyroid carcinoma.

The S100 proteins family is a group of calcium-binding proteins that is encoded by chromosome 1q21 and contains over 20 members with comparable amino acid structures^6^. These EF-hand proteins can interact with several target genes and are predominantly exist in a homodimeric form. S100 proteins perform a large range of functions, including regulating cell proliferation and invasion, interacting with the cytoskeleton interplays, and mediating transcription and inflammation^6,7^. Previous studies suggested that the S100 family exerts a crucial effects on the growth and metastasis of tumors by regulating the oncogenic microenvironment^6,8,9^. It has previously been reported that S100 proteins are expressed abnormally in plasma and tumor samples from patients with gastric, colon and pancreatic cancers^10–13^. S100A12, also called calgranulin C, is a calcium-binding protein that is composed of 92 amino acids and belongs to the S100 subfamily^14^. S100A12 expression is highly presented in neutrophils and low in lymphocytes and monocytes. The protein can be secreted and upregulated during acute and chronic infection. A previous report had found that S100A12 might exert anti-infective and anti-inflammatory effects in mammals^15^. Therefore, upregulated plasma levels of S100A12 are a novel biomarker for many inflammatory diseases^16^. In addition, S100A12 is expressed in multiple tumor cells, and contributes to mediating various vital cellular functions, including proliferation^17^,invasion, and migration^18^. It has been demonstrated that S100A12 is extensively expressed in the mucosa of oropharyngeal squamous cell carcinoma (OPSCC)and serves as a prognostic factor^19^. Moreover, S100A12 expression was related to pathological features, including the tumor grade and stage and lymph node metastasis status. Thierolf M *et al*. also demonstrated that S100A12 was highly expressed in colorectal cancer samples compared with normal colon samples^20^. In contrast, S100A12 mRNA and protein expression was reduced in gastric carcinoma (GC) tissues compared with normal control tissues and was correlated with TNM stage, tumor size and poor survival^21^. To date, multiple other members of the S100 protein family, such as S100A4^22^, S100A2, S100A6^23^, S100A9^24^ and S100A13^25^, have been shown to contribute to the development and prognosis of PTC. In addition, a targeted mass spectrometry approach was used to investigat S100 proteins in thyroid tumor samples, and the results were verified by western blotting. The results showed that S100A6, S100A11 and S100A13 were upregulated in PTC^26^. To date, there is no concrete evidence regarding the relationship between S100A12 expression and the pathological characteristics of PTC. However, S100A12 has been indicated to suppress proinflammatory and anti-inflammatory cytokines, suggesting that S100A12 could regulate proinflammatory and anti-inflammatory cytokines through the ERK signaling pathway activated^27^, which is also important in PTC Therefore, in this study, to investigate the contribution of S100A12, we examined its expression in human PTC samples by immunohistochemistry and western blotting. Meanwhile, explored its role in PTC cell proliferation migration, and invasion *in vitro* and *in vivo*.

## Materials and Methods

### Tissue specimen acquisition and immunohistochemistry

The study was performed with the approval of the ethics committee of First Affiliated Hospital of China Medical University, and informed consent was collected from all subjects with PTC whose tissue specimens were used in this study. The experiments were conducted according to the relevant guidelines and regulations. All patients had a clinical duration of less than 3 years and had been admitted to the hospital for a standard thyroidectomies between 2011 and 2013.Immunohistochemistry (IHC) was performed using an UltraSensitive^TM^ SP (Mouse/Rabbit) IHC Kit (Maixin Technology Co., Ltd, China). Rabbit polyclonal anti-S100A12(1:200, Abcam, Cambridge, UK), or rabbit polyclonal anti-Ki67 (1:100, Abcam, Cambridge, UK) were used. S100A12 expression was quantified using the following scoring method: 0 (negative), 1 (weakly positive), 2 (moderately positive), and 3 (strongly positive). The percentage of S100A12-positive cells was scored as follows: 0 (0% of cells stained), 1 (1%–25% of cells stained), 2 (26%–50% of cells stained), and 3 (50%–100% of cells stained). The intensity and percentage scores were multiplied, and the resulting scores were used to classify the samples into two groups: low expression (0–4.5) and high expression (4.5–9).

### Cell culture and transfection

Human PTC cell lines TPC1and K1 were kindly provided by Dr Wei Sun (The First Affiliated Hospital of China Medical University), who purchased them from Shanghai HonSun Biological Technology Co., Ltd. BHT101 and B-CPAP were purchased in Cell bank of Shanghai Chinese Academy of Sciences. The normal thyroid follicular epithelium cell line (nthy-ori3–1) was purchased from the American Type Culture Collection (ATCC), that was cultured in RPMI 1640 medium (Gibco, NY, USA). TPC1 cells were cultured in Dulbecco’s modified Eagle’s medium (DMEM, Gibco, NY, USA) containing 10% fetal bovine serum (FBS, Hyclone, UT, USA).K1 cells were cultured in DMEM:Ham’s F12 medium: MCDB105 (2:1:1) added with 2 mM glutamine and 10% FBS. BHT101 cells were cultured in DMEM Medium 500 ml, Glutamax (Gibco, NY, USA) 6 ml, FBS 125 ml. B-CPAP cells were cultured in RPMI Medium 1640 (Gibco, NY, USA) 87 ml, FBS 10 ml, NEAA (invitrogen, USA) 1 ml,Glutamax (invitrogen, USA), 1 ml Sodium, Pyruvate 100 mM Solution (invitrogen, USA) 1 ml. The cells were cultured under 5% CO_2_ atmosphere at 37 °C. The medium was changed every 2 days, and subcultured at 70–80% confluence. S100A12-RNAi lentiviral vectors were purchased from Shanghai GeneChem Company (Shanghai, China). The S100A12#1 sequence was 5′-CGACTTTCAAGAATTCATA-3′, the S100A12#2 sequence was 5′-GGATGCTAATCAAGATGAA-3′ and the shRNA control (shNC) sequence was 5′-TTCTCCGAACGTGTCACGT-3′.

### Western blot assay

Total protein was extracted using radioimmunoprecipitation (RIPA) buffer, and the total protein levels was confirmed with a bicinchoninic acid (BCA) assay (Beyotime, China). Twenty micrograms of protein was separated by 10% SDS-PAGE and was then transferred to polyvinylidene fluoride (PVDF) membranes. Membranes were incubated at 4 °C overnight with specific primary antibodies, namely: rabbit polyclonal anti-p-ERK, rabbit polyclonal anti-t-ERK, rabbit polyclonal anti -CyclinD1, rabbit polyclonal anti-CDK4 (all 1:1000, Cell Signaling Technology, Boston, MA, USA) rabbit polyclonal anti-S100A12 (1:1000, Abcam, Cambridge, UK), and mouse polyclonal anti-GAPDH (1:5000, Santa Cruz, USA) The membranes were followed by incubation with a goat anti-rabbit secondary antibody or goat anti-mouse secondary antibody (1:5000, Origene Co., Ltd. Beijing, China). The reactions were examined by an enhanced chemiluminescence assay.

### MTT assay and colony formation assays

Cell viability was assessed using 3-(4,5-dimethylthiazol-2-yl)-2,5-diphenyltetrazolium bromide (MTT) (Sigma Aldrich, St. Louis, MO, USA). Cells were seeded at 1000 cells/well in 96-well plates at 1 d, 2 d and 3 d after treatment. Samples were incubated in medium containing 100 mg/0.1 ml of MTT for 4 h. Crystals were dissolved in DMSO after incubation for 4 hours in medium containing MTT. The optical density (OD) was counted at 490 nm using by a spectrophotometer. A plate colony formation assay was performed to assess the cells’ colony formation ability. Cells were seeded into 6 well plates at a density of 500–1000 cells/well and cultured for 14 days. Cells were then fixed in 4% paraformaldehyde and stained with crystal violet solution. The number of colonies was counted in 3 different wells and the mean value was calculated. The assays were performed in triplicate.

### Wound healing assay

The cells were seeded in 6-well tissue culture plates and grown to 80–90% confluence. A scratch was made using a 10 μl pipette tip and the result of the wound healing experiment was photographed at 0 h and 24 h. The wound healing rate was quantitatively evaluated using Olympus CellSens Dimension software. Each assay was performed in triplicate.

### Transwell migration and invasion assays

Transwell assays were performed to investigate the cells’ migration and invasion. Cell invasion was detected by Transwell chambers (Corning, Corning, NY, USA) coating with Matrigel (60 μl, 1:6 dilution; BD Biosciences). 1.2 × 10^5^ cells/200 μL, FBS-free medium, were added into the upper chamber, and culture medium containing 10% FBS was seeded to the lower chamber. After 24 hours, cells were immobilized, stained and the numbers of invasion cells were calculated by microscope. For the migration assays, the upper chambers without Matrigel and the subsequent steps were the same as described invasion assays.

### Flow cytometric assays

Cells were collected and fixed in 70% ice-cold ethanol. Before cell cycle analysis, the cells were stained using propidium iodide (PI) (50 μg/ml, Sigma Aldrich) containing RNase (8 μg/ml, Sigma Aldrich) at 37 °C for 30 minutes. Cell cycle distribution was quantified using a flow cytometer.

### Nude mouse xenograft model

Nude mouse were purchased from Animal Department of China Medical University. The procedures were conducted aseptically in according to the institutional manual of the University Committee on the Use and Care of Animals at China Medical University. Approximately 1 × 10^7^ cells in 200 µl PBS were injected subcutaneously into the right subaxillary region of each mouse. The tumor volume was calculated weekly. After 4 weeks, mice were sacrificed, and tumors were collected for H&E staining and immunochemical studies.

### Statistical analysis

Data are shown as means ± SD of at least three independent experiments. Statistical evaluation of the data was conducted by using an unpaired t-test, and ANOVA followed by Tukey’s post hoc test and the Mann-Whitney U test. All statistical analyses were was conducted by SPSS 19.0 software (IBM Corporation, Armonk, NY, USA). Differences were taken for statistically significant at *P* < 0.05.

### Compliance with ethical standards

This study was conducted with the approval of the ethics committee of First Affiliated Hospital of China Medical University, and informed consent was obtained from all the patients with PTC whose tissue specimens were used in this study. All methods was performed in accordance with the relevant guidelines and regulations.

All animals procedures were performed aseptically in accordance with the institutional guidelines of the University Committee on the Use and Care of Animals at China Medical University. All animal experiments conformed entirely to the National Animal Care and Ethics Committee guidelines. The experimental protocols were approved by Animal Care and Use Committee of China Medical University.

## Results

### S100A12 is overexpressed in PTC and associated with clinicopathological features

**Figure 1:**
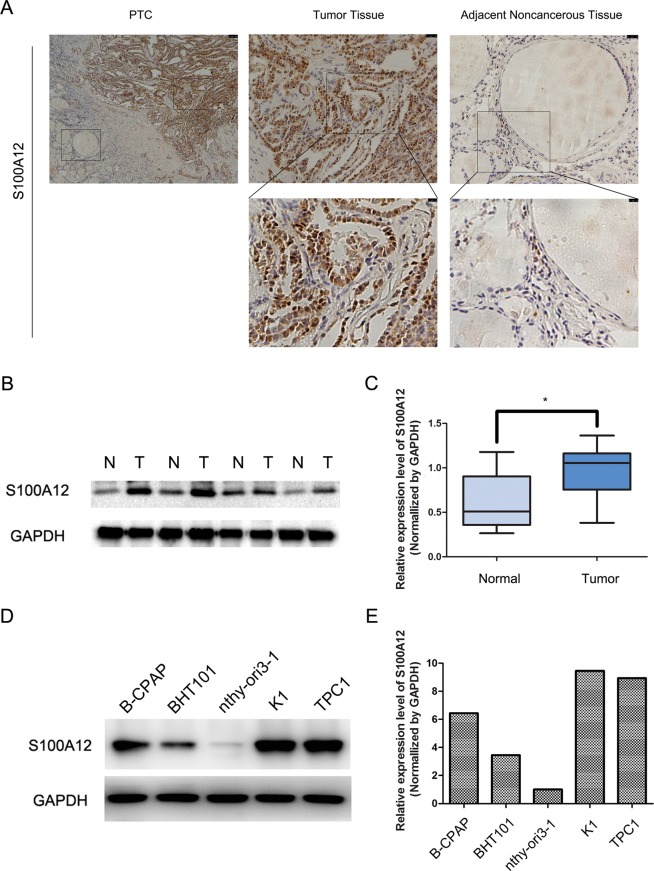
Expression of S100A12 in PTC tissue samples. (**A**) Immunohistochemical analyses of PTC tissues and adjacent noncancerous tissues for S100A12. (**B**) Western blotting of S100A12 expression in paired PTC tissues(T) and their adjacent noncancerous tissues(N). (**C**) S100A12 protein level in 13 paired PTC tissues and adjacent noncancerous tissues, GAPDH was detected as a loading control. ^*^P < 0.05. (**D**,**E**) Western blotting of S100A12 expression in TPC1, K1, BHT101, B-CPAP and nthy-ori3-1 cell lines.

We examined the protein expression of S100A12 in 109 cases of PTC. The S100A12 protein was mainly localized in the nucleus and cytoplasm of cancerous cells and negative S100A12 expression was detected in the adjacent normal tissue (Fig. 1A). To further determine the expression pattern of S100A12 in human PTC tissues, we compare the protrein levels of S100A12 in 13 pairs of PTC tissues through western blot assay. The results showed that the expression of S100A12 was significantly higher in the PTC samples than in the matched normal samples (*P < 0.05, Fig. 1B,C). As shown in Table 1, overexpression of S100A12 was significantly correlated to the tumor size (*P < 0.05), tumor TNM stage (*P < 0.05) and lymph node metastasis (*P < 0.05). No correlations were found with the patients’ sex, or age. These findings indicate that S100A12 expreesion was highly increased in PTC samples than in matched normal tissues and was associated with the tumor size, tumor stage and lymph node metastasis in PTC.

**Table 1:** Clinicopathologic features of 109 thyroid carcinomas patients.

characteristics	Expression of S100A12		*P* value
	Low	High	
	Number = 57	Number = 52	
**Gender**			
Male	15	17	0.465
female	42	35	
**Age (years)**			
<55	45	38	0.473
≥55	12	14	
LNM			
Yes	25	39	0.039*
No	32	13	
**TNM Stage**			
I	49	17	<0.001*
II	4	9	
III	2	4	
IV	2	22	
**Tumor size (cm)**			
<2	24	8	0.002*
≥2	33	44	

LNM: lymph node metastasis.

Statistical analyses were performed by the Pearson χ^2^ test. *p < 0.05 was considered significant.

### S100A12 regulates the tumorigenic behavior of PTC cells *in vitro*

**Figure 2:**
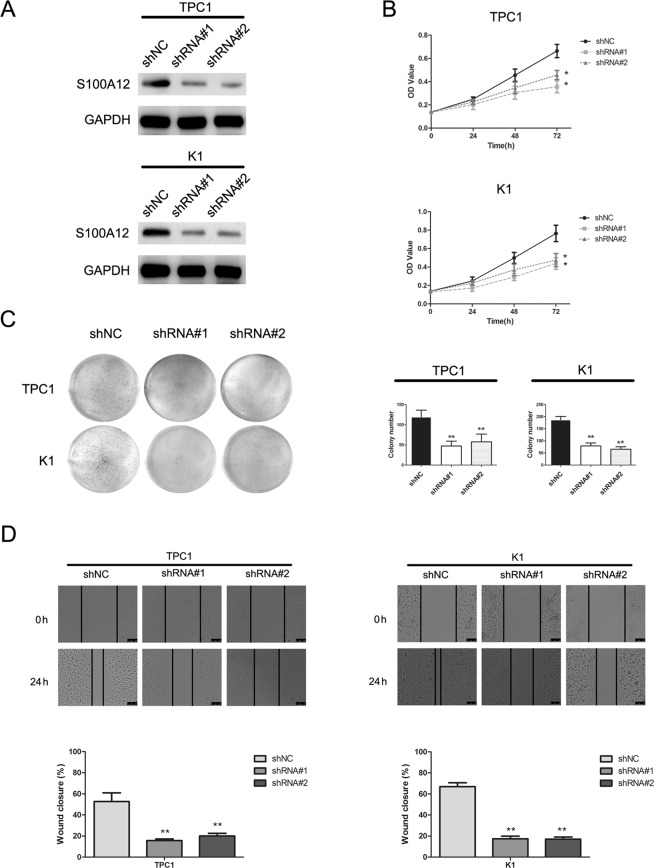
Knockdown of S100A12 inhibits PTC cells growth. (**A**) Western blot assay evaluated the S100A12 protein after transfected with shRNA#1 and shRNA#2 in TPC1 and K1 cells. (**B**) MTT assay showed that downregulation of S100A12 decreased the growth rate of PTC cells. ^*^P < 0.05. (**C**) Silencing S100A12 significantly reduced the colony number in the colony formation assay. ^**^P < 0.01. (**D**) S100A12 knockdown inhibits migration of TPC1and K1 cells. All data are demonstrated as a mean ± SD of three independent experiments. **P < 0.01.

**Figure 3:**
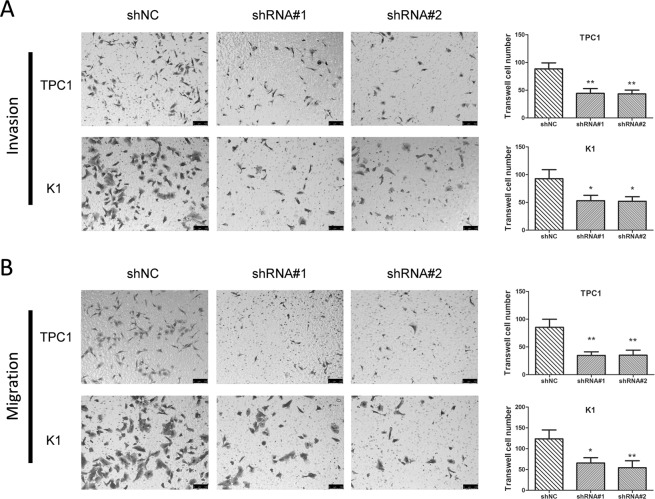
Silencing S100A12 retarded cellular migration and invasion in PTC cells. (**A**) Knockdown of S100A12 inhibits TPC1 and K1 invasion cells. ^*^P < 0.05, **P < 0.01. (**B**) Representative of image of TPC1 and K1 cell migration, and quantitative results of migration cell numbers. ^*^P < 0.05, ^**^P < 0.01.

We examined S100A12 expression in 4 PTC cell lines (TPC1, K1, BHT101 and B-CPAP) and a normal thyroid follicular epithelium cell line (nthy-ori3-1) through western blot assay, both TPC1 and K1 expressing the highest level (Fig. 1D,E). To investigate the function of S100A12 in PTC cell lines, we selected TPC1 and K1 cells and downregulated S100A12 via the transfection of two S100A12-specific shRNAs (shRNA#1, shRNA#2) transfection. The transfection efficiency was examined via a western blot assay (Fig. 2A), and the proliferation of PTC cells were assessed by an an MTT and a colony formation assay. Both MTT and colony formation assay showed that silencing of S100A12 significantly reduced the proliferation of PTC cells (Fig. 2B,C; ^*^P < 0.05, ^**^P < 0.01). Furthermore, wound healing assay demonstrated that downregulation of S100A12 can decrease the migration rate of PTC cells after 24 h of shRNAs transfection (Fig. 2D). The results of the Transwell assays further verified that S100A12 silencing can suppress the invasion and migration of PTC cells (Fig. 3A,B; ^*^P < 0.05, ^**^P < 0.01). Taken together, these results indicated that S100A12-silenced decrease PTC cells proliferation, migration and invasion.

### S100A12 knockdown induces G0/G1 phase accumulation of PTC cells

**Figure 4:**
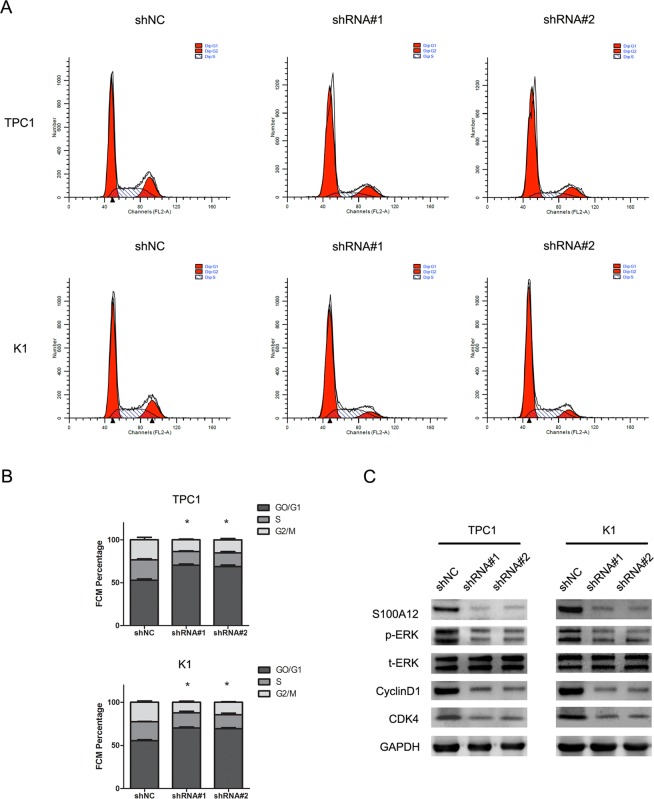
S100A12 knockdown induced PTC cells G0/G1 phase accumulation. (**A**) Effect of S100A12 silenced on cell cycle progression of the TPC1 and K1 cells, as determined by flow cytometry. (**B**) Statistical results of flow cytometry.*P < 0.05. (**C**) Western blot analysis of the expression levels of p-ERK,t-ERK,CyclinD1 and CDK4, in TPC1 and K1, after silencing S100A12.

As knockdown of S100A12 inhibited cell proliferation, we further investigated the effect of S100A12 in the process of cell cycle. This analysis of TPC1 and K1 cells was conducted by flow cytometry assay. These results showed a significant up-regulation in the G0/G1 phase population after S100A12 silencing (Fig. 4A; *P < 0.05). To determine how S100A12 affects PTC cell growth, we also analyzed the protein levels of p-ERK, t-ERK, CyclinD1, and CDK4 in cells transfected with shRNA#1 and shRNA#2, which showed a remarkable down-regulation in the levels of p-ERK, CyclinD1 and CDK4. The results showed no significant alternation in the protein levels of t-ERK (Fig. 4B).

### S100A12 regulates the tumorigenic behavior of PTC cells *in vivo*

**Figure 5:**
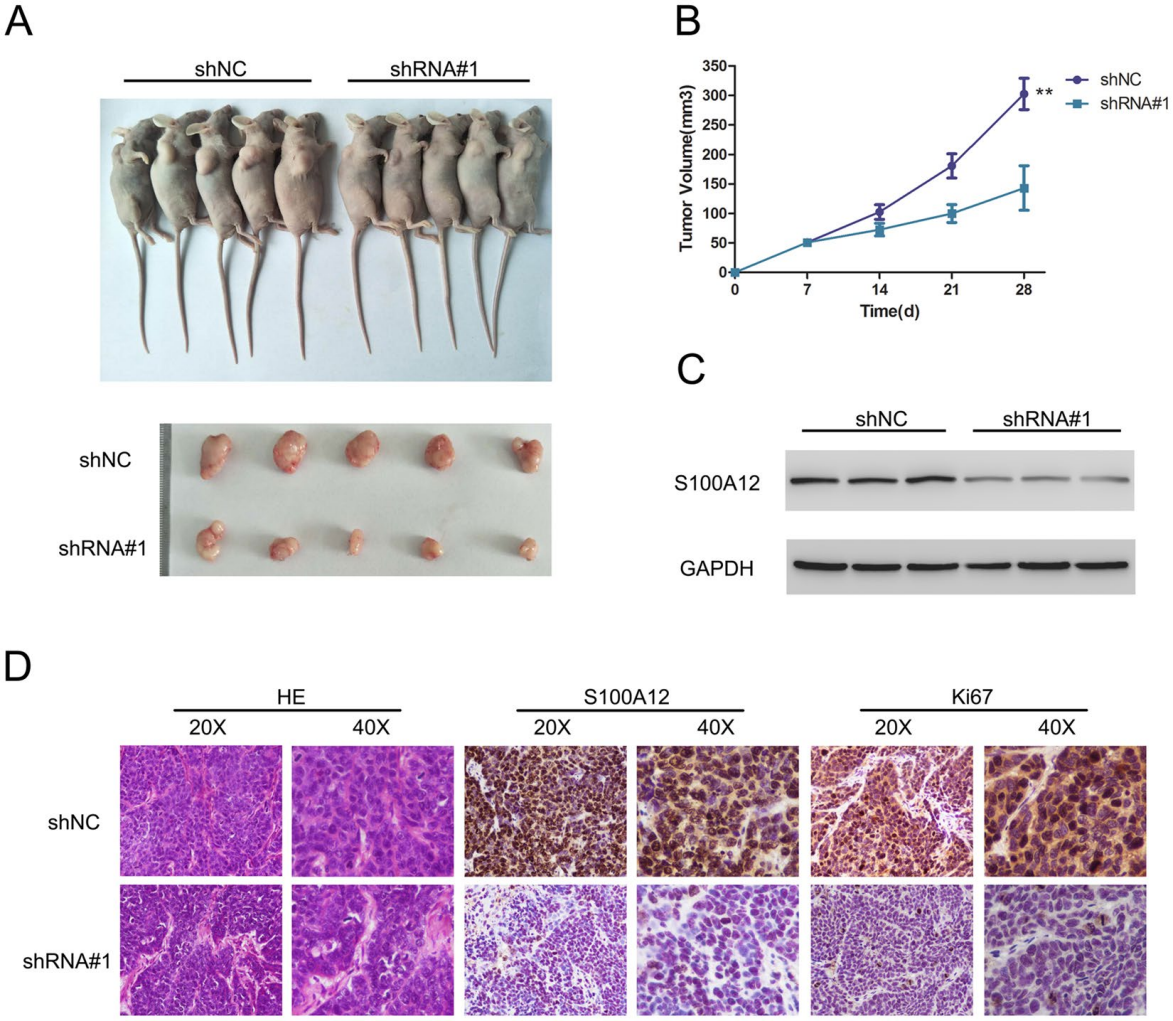
Stable knockdown of S100A12 suppressed tumor growth *in vivo*. (**A**) Images of nude mouse graphs and the excised tumors from these mouse at 28 days after injection with shRNA#1-transfected cells and shNC-transfected cells. (**B**) Tumor volumes were measured every 7 days. (**C**) Western blot assay detects the expression of S100A12 in excised tumors. (**D**) The results of hematoxylin and eosin (H&E) staining and immunohistochemical (IHC) staining of S100A12 and Ki67 in the tumors.

To further investigate whether S100A12 knockdown suppresses tumorigenesis *in vivo*, we established a xenograft model using TPC1 cells to assess the role of S100A12 RNAi *in vivo*. The results showed that tumor growth was significantly decreased in nude mice injected with shRNA#1- transfected cells compared to mice injected with vector-transfected (shNC) cells (Fig. 5A,B). Western blot assays confirmed that the protein levels of S100A12 were decreased in tumor cells from the shRNA#1 group compared with tumor cells from the shNC group (Fig. 5C). Consistent with this result, the nuclear expression of S100A12 and Ki67 proteins was decreased in tumor cells after S100A12 silencing.

## Discussion

The present study found by western blot and immunohistochemistry that S100A12 expression is significantly increased and is associated with clinicopathological characteristics in human PTC samples. In addition, upregulation of S100A12 was significantly associated with tumor size, tumor-node-metastasis (TNM) stage and lymph node metastasis (LNM). Furthermore, silencing S100A12 in two PTC cell lines, namely, TPC-1 and K1 suppressed cell proliferation, invasion and migration. Additionally, silencing S100A12 also induced cell cycle arrest and ERK signaling. The *in vivo* study also found that silencing S100A12 reduced the aggressive growth of tumor cells in xenografted mice. This study suggested that S100A12 might serve as a novel diagnostic biomarker in PTC.

The S100 protein subfamily has been demonstrated to mediate multiple cellular processes, including cell proliferation and growth, cell cycle advancement and protein phosphorylation^7^. Currently, it has been reported that S100 proteins are related to a number of human diseases, such as inflammatory disorders and different kinds of cancers^17^. Accordingly, the correlation between S100 proteins and PTC has have been investigated in various studies. Some members of this protein family might be used as therapeutic targets in PTC. It has been previously reported that the S100 proteins S100A2, S100A4, S100A6, and S100A9 are upregulated in thyroid cancer and that these subfamily members are involved in the progression of cancer in different ways^22–24^. Moreover, Zhong *et al*. found that S100A13 was upregulated in thyroid cancer tissues and that overexpression of S100A13 promoted cell growth in an *in vivo* xenograft model. In addition, silencing S100A13 resulted in the inhibition of cancer growth and invasion in thyroid cancer cell lines^25^. Furthermore, S100A4 was abundantly expressed in thyroid cancers and strongly associated with LNM and poor prognosis^28^. Targeting S100A4 may decrease the invasion and metastasis in PTC. Moreover, extracellular S100A4 can induce ERK signaling pathway in thyroid cancer^29^. However, limited sources about the role of S100A12 in PTC have been found. Multiple studies have reported that S100A12 is involved in cancer-related processes and positively correlated with the prognosis of cancer. It has been reported that the serum levels of S100A12 were highly increased in colorectal cancer patients compared with healthy subjects^20^. It was also implied that S100A12 could be used as a novel prognostic biomarker to determine recurrence and metastasis in the early phase during hepatectomy^30^. In addition, S100A12 has been found to be markedly decreased in gastric carcinoma tissues and related to pathological characteristics, tumor size, TNM stage and tumor cell invasion and differentiation^21^. Funk *et al*. demonstrated that upregulation of S100A12 was a prognostic factor in oropharyngeal squamous cell carcinoma^19^, while Li *et al*. proved that the decreased expression of S100A12 in tumor cells may contribute to the tumorigenesis of gastric cancer^21^. This finding was in contrast with our results. This may be due to the discrepancy of tissue types. The transcriptional level analysis also revealed a reduction in the overall expression of S100A12 mRNA in tumor tissue compared to normal mucosa, which showed that the mechanism of S100A12 downregulation in gastric cancer may occur at the transcriptional level, not at the protein levels, as our research indicated. In accordance with previous studies, we found that S100A12 protein expression was dramatically increased in human PTC samples. These results indicated that S100A12 may function as an oncogene in the development of PTC.

In our study, we also analyzed the role of S100A12 in PTC cell lines, namely, TPC-1 and K1. The results of wound healing and Transwell assays showed that the migration and invasion of PTC cells were reduced by knockdown of S100A12. Moreover, the colony formation assay also showed that the proliferation of PTC cells *in vitro* was downregulated by S100A12 silencing. These *in vitro* results demonstrated that silencing S100A12 reduced the progression of tumor cells.

Dysregulation of the cell cycle is a general mechanism of tumorigenesis, and the dysfunction of this process plays a vital role in carcinoma pathogenesis^31^. In the present study, we examined the role of S100A12 in the cell cycle in thyroid cell lines and found that silencing S100A12 induces the process of cell cycle arrest and increases the population of t G0/G1 phase cells. We also found that knockdown of S100A12 reduced CyclinD1 and CDK4 protein expression in the TPC1 and K1 cell lines. Dysfunction of the cell cycle is a major characteristic of tumorigenesis, which implies that cell cycle arrest in G1 phase or CDK expression may contribute to tumor progression^32^. In addition, it has been previously demonstrated that CyclinD1 and CDKs are major regulators in the G1 phase of the cell cycle^33,34^. CyclinD1 is frequently dysregulated in carcinoma cells and functions as a biomarker for cancer and disease progression^35^. It has been previously reported that the expression of CyclinD1 and CDK4 results in multiple cancer hallmarks by promoting the proliferation of cancer cells^36^. Moreover, overexpression of CyclinD1 promotes the tumor growth and metastasis in PTC^37^. The results herein suggest that the S100A12-related G1/S transition may be involved in the inhibitory effect of S100A12 on the development of thyroid cancer.

To discover the potential signaling pathway contributing to the regulatory role of S100A12 in thyroid cell progression, we examined the levels of phosphorylated ERK and total ERK in both thyroid cancer cell lines. The ERK signaling pathway is known to play a crucial role in tumor cell development^38^. The ability of S100A12 to stimulate ERK1/2 activity has been widely demonstrated, but there are many possible ways to activate ERK1/2^27^. First, S100A12 could act from inside cancer cells to activate ERK1/2 pathways. S100A12 expression in the livers of septic rats was positively related to the mRNA expression of ERK1 and ERK2, showing that S100A12 can regulate the ERK signaling pathway in govening the serum inflammatory response in septic rats^39^. Additionally, S100A12 can be a receptor for the RAGE ligand, and advanced glycation end products (AGEs) are related to the activation of p21ras, which is correlated with the ERK pathway^40^, suggesting that S100A12 could regulate the ERK signaling pathway. However, the central mechanisms connecting S100A12 and ERK1/2 pathway activation in this study were unclear, and further studies need to confirm the mechanisms underlying ERK1/2 activation by S100A12 in PTC progression. Our results found that silencing S100A12 significantly reduced the protein level of phosphorylated ERK. S100A12 can activate the migration of human aortic smooth muscle cells via the ERK signaling pathway^41^. ERK is also reduced by S100A12 in chronic airway disease^42^. In addition, we supplement the experiment that using SCH772984 to inhibit ERK activity in K1 and tpc1 cells, which was consistent with silencing S100A12. In general, our findings indicate that the contribution of S100A12 to the cell proliferation, migration and invasion of TPC-1 and K1cells might be regulated via the ERK signaling pathway.

To further investigate the mechanism underlying the effect of S100A12 shRNA treatment in the xenograft mouse model, we analyzed tumor growth by H&E staining and examined S100A12 and Ki67 expression in tumor sections by IHC. Tumors with.S100A12 silencing exhibited decreased growth and reduced expression of S100A12 and Ki67, suggesting that proliferation was highly inhibited to S100A12 shRNA treatment as compared with vehicle treatment. These results indicate that silencing S100A12 can suppress PTC cells *in vivo*.

## Conclusion

In general, this study is the first to investigate S100A12 expression in thyroid cancer. Downregulation of S100A12 could inhibit the growth of thyroid cancer. These results provide crucial evidence of the effect of S100A12 on theprogression of thyroid carcinoma, and suggest that S100A12 to be is a novel biomarker for the diagnosis of thyroid cancer.

## Supplementary information

Supplementary information.
