# Supplementary material for: S100A12 is a promising biomarker in papillary thyroid cancer
Source: Sci Rep. 2020 Feb 3;10:1724. doi: 10.1038/s41598-020-58534-1 (PMC6997206; doi:10.1038/s41598-020-58534-1)
Supplement: Supplementary file 1 — Supplementary information. [file 41598_2020_58534_MOESM1_ESM.docx]

**S100A12 is a promising biomarker in papillary thyroid cancer**

Xiaojie Wang ^1^, Zhenxiang Sun^1^ , Wei Tian^1^, Chenghao Piao^2^, Xiaochen Xie^3^, Jin Zang^1^ ,Shiqiao Peng^3^, Xiaohui Yu^3^, Yiwei Wang^1，*^

1, Department of anatomy, Shenyang Medical College, Huanggu District, Shenyang City, Liaoning Province, P. R. China, 110034

2, Department of Radiology, The Second Affiliated Hospital of Shenyang Medical College, Shenyang City, Liaoning Province, P.R. China, 110035

3, Department of Endocrinology and Metabolism, Institute of Endocrinology, Liaoning Provincial Key Laboratory of Endocrine Diseases, The First Affiliated Hospital of China Medical University, China Medical University, Shenyang, Liaoning, P.R. China, 110001

**Running title:** S100A12 in Papillary thyroid carcinoma.

* Corresponding author: Yiwei Wang

Department of human anatomy, Shenyang Medical College, Huanggu District, Shenyang City, Liaoning Province 110034, P. R. China.

Phone: 0086 18624005671

Fax: 0086 024 62216816

Email address: wangyiwei_symc@163.com

**Inhibition of the tumorigenesis of PTC cells by SCH772984**

SCH772984, dissolved in dimethyl sulfoxide (DMSO) as a 0.25 µM solution, was used to inhibit the activity of p-ERK. We performed MTT assays and colony formation assays to evaluate the proliferation of PTC cells (K1 and TPC1). We observed dramatic inhibition of PTC cell growth after treatment with SCH772984 compared with control cell. (Figure SL A and B; * *P* <0.05, ** *P* <0.01, *** *P* <0.001). Furthermore, the transwell assay results showed that the SCH772984 slowed the migration and invasion of PTC cells (Figure SL C; ** *P* <0.01). Taken together, these results indicate that ERK inhibition is consistent with S100A12 silencing in these PTC cell lines.


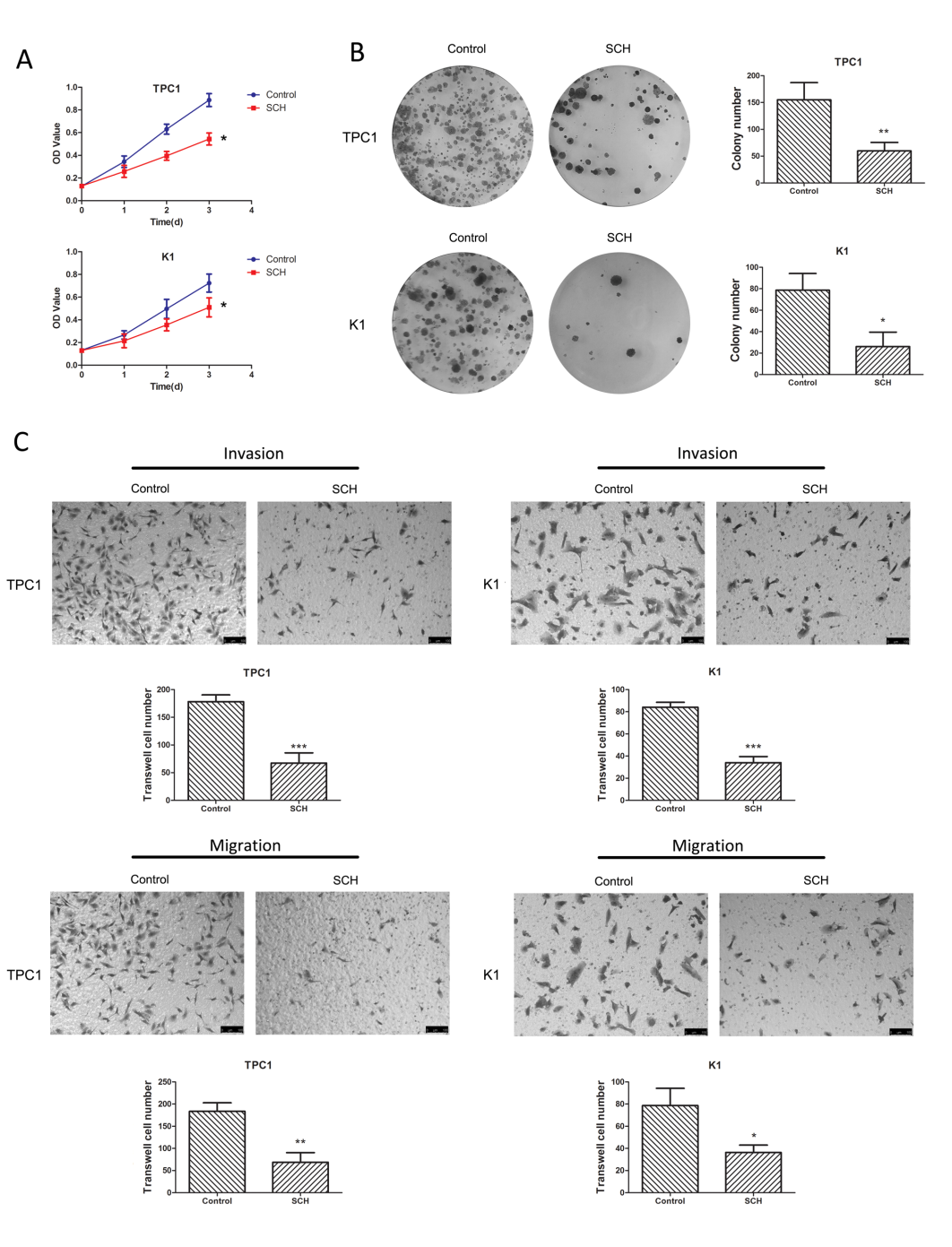


ERK inhibitor SCH772984 treatment decreases proliferation， invasion and migration of PTC cell lines.

1. TPC1 and K1 cells were treated with DMSO, the solution of SCH772984 (Control ) , or SCH772984 0.25 μM (SCH) for 1d, 2d and 3d. Cell viability was measured by a MTT assay. * *P* < 0.05.
2. Colony formation assay. The colony number was inhibited by SCH772984. * *P* < 0.05, * **P* < 0.01
3. The PTC cells invasion and migration were evaluated through Transwell assays. * *P* < 0.05, * **P* < 0.01, * * * *P* < 0.001.
